# Safety and pharmacokinetics of paclitaxel and the oral mTOR inhibitor everolimus in advanced solid tumours

**DOI:** 10.1038/sj.bjc.6604851

**Published:** 2009-01-06

**Authors:** M Campone, V Levy, E Bourbouloux, D Berton Rigaud, D Bootle, C Dutreix, U Zoellner, N Shand, F Calvo, E Raymond

**Affiliations:** 1 Department of Medical Oncology, Centre René Gauducheau, Nantes, Cedex 44805, France; 2 Department of Medical Oncology, Hôpital Saint Louis AP-HP, Paris, Cedex 10, France; 3 Novartis Pharma AG, Basel, 4002 Switzerland; 4 Hôpital Beaujon, Clichy, France

**Keywords:** RAD001, everolimus, paclitaxel, phase I, drug interaction, combination chemotherapy

## Abstract

Everolimus displays antiproliferative effects on cancer cells, yields antiangiogenic activity in established tumours, and shows synergistic activity with paclitaxel in preclinical models. This study assessed the safety and the pharmacokinetic interactions of everolimus and paclitaxel in patients with advanced malignancies. Everolimus was dose escalated from 15 to 30 mg and administered with paclitaxel 80 mg m^−2^ on days 1, 8, and 15 every 28 days. Safety was assessed weekly, and dose-limiting toxicity (DLT) was evaluated in cycle 1. A total of 16 patients (median age 54.5 years, range 33–69) were entered; 11 had prior taxane therapy for breast (*n*=5), ovarian (*n*=3), and vaginal cancer (*n*=1) or angiosarcoma (*n*=2). Grade 3 neutropenia in six patients met the criteria for DLT in two patients receiving everolimus 30 mg weekly. Other drug-related grade 3 toxicities were leucopenia, anaemia, thrombocytopenia, stomatitis, asthenia, and increased liver enzymes. Tumour stabilisation reported in 11 patients exceeded 6 months in 2 patients with breast cancer. Everolimus showed an acceptable safety profile at the dose of 30 mg when combined with weekly paclitaxel 80 mg m^−2^, warranting further clinical investigation.

The mammalian target of rapamycin (mTOR) is an important kinase downstream in the PI3-kinase/Akt signalling pathway. It modulates signals from growth factors and intracellular sensors of nutrients and hypoxia to control translation of proteins involved in cellular growth, proliferation, and responses to nutrient deprivation and hypoxic stress, resulting in the stimulation of angiogenesis, facilitation of G_1_–S transition of the cell cycle, and inhibition of radiation or cytotoxic drug-induced apoptosis in a variety of human tumours (Faivre *et al*, 2006). Cancer cells commonly have mutations and/or overexpression of several signalling molecules, resulting in the activation of mTOR and its substrates S6K1 and 4E-BP1. For example, the HER2 overexpression observed in about one-third of breast cancers is associated with activation of PI3-kinase/Akt/mTOR signalling (Bacus *et al*, 2002; Zhou *et al*, 2004), resistance to stress-induced apoptosis (Bacus *et al*, 2002), and tumour aggressiveness (Cobleigh *et al*, 1999; Zhou *et al*, 2004). Activation of the PI3-kinase/Akt/mTOR signalling pathway may be inhibited by rapamycin, which blocks mTOR activation, resulting in the inhibition of tumour growth (see reviews by Faivre *et al*, 2006; Georgakis and Younes, 2006).

Everolimus (RAD001) is an oral mTOR inhibitor derived from rapamycin, which blocks the activation of S6K1 and 4E-BP1, thereby inhibiting cell growth, proliferation, and G_1_–S transition, and inducing apoptosis (Boulay *et al*, 2004). Everolimus displays direct effects on growth and proliferation of cancer cells and inhibits angiogenesis by preventing the proliferation of endothelial cells in human tumour xenografts (Beuvink *et al*, 2001; Lane *et al*, 2003). In single-agent phase I studies of everolimus performed in patients with advanced cancers, a safe toxicity profile along with evidence of sustained tumour stabilisation at weekly doses of 10–70 mg was shown (O'Donnell *et al*, 2008; Tabernero *et al*, 2008). Dose-limiting toxicities (DLTs) observed at 70 mg included grade 3 stomatitis, neutropenia, and hyperglycaemia (Tabernero *et al*, 2008). Consistent inhibition of S6K1 activity in peripheral blood mononuclear cells was seen with doses ⩾20 mg per week and was sustained for at least 7 days at doses ⩾20 mg per week (O'Donnell *et al*, 2008). Furthermore, immunohistochemical analysis in paired tumour tissue biopsies taken before and during weekly dosing (20, 50, or 70 mg) confirmed inhibition of S6K1 at doses ⩾20 mg (Tabernero *et al*, 2008).

Paclitaxel remains a standard treatment for a variety of cancers, including lung, breast, head and neck, and ovary, and may be administered weekly to reduce haematological toxicity either alone or in combination with other anti-tumour agents on an every-week (Green *et al*, 2005; Le *et al*, 2006; Schuette *et al*, 2006; Yamamoto *et al*, 2006) or a weekly for 3 weeks monthly schedule (Perez *et al*, 2005; Yeh *et al*, 2005; Ramalingam *et al*, 2006; Stathopoulos *et al*, 2007). Several tumour types that display sensitivity to paclitaxel also show activation of the PI3-kinase/Akt/mTOR signalling pathway (Shayesteh *et al*, 1999; Hu *et al*, 2000; Perez-Tenorio *et al*, 2002; David *et al*, 2004; Samuels *et al*, 2004; Saal *et al*, 2005). Paclitaxel resistance appears multifactorial, involving overexpression of P-glycoprotein, mutation of *β*-tubulin, and defects of apoptosis (Yusuf *et al*, 2003). There is also consistent evidence that the PI3-kinase/Akt/mTOR signalling pathways may be associated with resistance to taxanes and other drugs acting on microtubules. In cancer cells, inhibition of the PI3-kinase/Akt/mTOR signalling pathway (Hu *et al*, 2002; Yu *et al*, 2005), including inhibition of mTOR with rapamycin, was shown to counteract Akt-mediated resistance to drugs inhibiting tubulin (Vanderweele and Rudin, 2005) and to restore apoptosis (Faried *et al*, 2006).

*In vivo* combinations of everolimus with paclitaxel showed additive and synergistic effects in tumour models but with a schedule effect, in that administration of everolimus 1 day before or after paclitaxel reduced anti-tumour activity (O'Reilly *et al*, 2003). These preclinical data served as a rationale to further investigate the safety and potential pharmacokinetic interactions of everolimus in combination with paclitaxel in patients with tumours potentially sensitive to paclitaxel-based chemotherapy. To avoid effects related to scheduling and accumulation, everolimus and paclitaxel were administered together on the first day of each week, and in week 4 of each cycle treatment, it was omitted to allow recovery of cells from mTOR inhibition.

## Materials and methods

This open-label dose-escalation study was approved by the French National Ethics Committee and was conducted in accordance with the Declaration of Helsinki Principles and Good Clinical Practice. A signed informed consent was required of each patient.

### Eligibility criteria

Eligible patients were at least 18 years of age with pathologically confirmed locally advanced or metastatic cancer refractory to or unsuitable for standard chemotherapy, but they were potentially able to benefit from paclitaxel chemotherapy. Life expectancy was ⩾6 months with a World Health Organization (WHO) performance status of 0–2. Patients also had adequate bone marrow (absolute neutrophil count ⩾1.5 × 10^9^ l^−1^, platelets ⩾100 × 10^9^ l^−1^, haemoglobin >10 g per 100 ml), liver function (serum transaminase ⩽3 times upper limit of normal (ULN), serum albumin and bilirubin within reference ranges), and kidney function (creatinine ⩽1.5 times ULN).

Patients were ineligible if they had primary or metastatic central nervous system cancer, a history of HIV seropositivity, or active bleeding diathesis; were taking oral anti-vitamin K medication (except low-dose coumadin); had uncontrolled infection, impaired gastrointestinal function, or disease that might alter absorption of everolimus; or had received an investigational drug within the previous 30 days. Women who were pregnant, breast-feeding, or able to conceive but unwilling to practice effective contraception, and patients with known Cremophor allergy were also excluded.

### Study drug administration and monitoring

Everolimus (RAD001; Novartis Pharmaceuticals, Basel, Switzerland) and paclitaxel (Bristol-Myers Squibb Pharmaceuticals Ltd, New York, NY, USA) were administered on days 1, 8, and 15 on an every-28-day cycle for up to 6 cycles. Everolimus was given orally immediately before paclitaxel, which was administered by intravenous infusion over 60 min. Patients receiving at least one dose of study drug medications were evaluated weekly for safety using the National Cancer Institute Common Toxicity Criteria version 2.0. Safety assessments consisted of clinical examinations and scheduled laboratory evaluations and electrocardiograms. The paclitaxel–everolimus combination was interrupted on the occurrence of any grade 3 non-haematological toxicity or grade ⩾2 thrombocytopenia or neutropenia until recovery to grade ⩽1. Treatment with the combination was discontinued on any grade 4 toxicity or failure to return to grade ⩽1 within 2 weeks. Prophylaxis of emetic and allergic reactions to paclitaxel using ondansetron, granisetron, and steroids was allowed. The concomitant use of any drugs that may interfere with cytochrome CYP450-3A was discouraged. After paclitaxel discontinuation at the completion of six cycles, everolimus could be maintained weekly until tumour progression. Anti-tumour activity was assessed using Response Evaluation Criteria in Solid Tumors.

### Dose escalation and definition of DLT

Because everolimus could potentially increase exposure to paclitaxel and, at higher doses than used in this study, had shown significant haematological toxicity, the safety of the combination was explored in patients in whom the standard weekly dose of paclitaxel 80 mg m^−2^ was combined initially with one-half the single-agent recommended dose of everolimus (15 mg weekly, dose level 1) and then escalated to 30 mg weekly (dose level 2). Dose-limiting toxicity was defined as occurring during the first 28-day cycle, suspected of being drug related, and included any grade 4 toxicity, any grade 3 non-haematological toxicity despite preventive therapy, or grade ⩾2 neutropenia, or thrombocytopenia failing to revert to grade ⩽1 within 2 weeks. Three patients were entered per dose level, with expansion to six patients if one of three patients experienced DLT. If DLT occurred in none of three or no more than one of six patients in dose level 1, doses would be escalated to dose level 2. Dose-limiting toxicity in more than one of three or more than two of six patients would be considered unacceptable. After the highest well-tolerated dose of everolimus (15 or 30 mg) in combination with paclitaxel was established, the size of this cohort would be expanded to 12 patients to obtain additional safety information on cumulative toxicity. With a sample size of 12 patients, the expanded cohort had a probability of 72 and 99% of detecting any toxicity that occurs with an incidence of 10 and 30%, respectively.

### Pharmacokinetic measurements

In week 1 of cycle 1, blood samples were taken together to measure pharmacokinetic parameters of both everolimus and paclitaxel. At cycle 2, everolimus was withheld on day 1, allowing the determination of blood levels of paclitaxel alone, without interference from everolimus, which shows a terminal half-life of about 30 h as a single agent. Pharmacokinetic parameters of everolimus were determined in week 2 of cycle 2, 1 week following the last administration of paclitaxel, which displays a terminal half-life of about 10 h as a single agent. For paclitaxel, sampling was carried out before and at the end of the infusion and at 15 and 30 min, and 1, 2, 4, 9, 24, 36, 48, and 96 h after completion of the infusion. For everolimus, sampling was carried out predose and 1, 2, 4, 6, 12, 24, 48, 96, and 168 h after administration. Everolimus was measured in whole blood by liquid chromatography–mass spectrometry after liquid extraction, its lower limit of quantification being 0.368 ng ml^−1^. Paclitaxel was measured in plasma by high-pressure liquid chromatography with ultraviolet detector, lower limit of quantification being 10 ng ml^−1^. Pharmacokinetic parameters were derived by standard non-compartmental methods of analysis (WinNonlin Pro 5.0). The measured parameters included the maximum plasma concentration (*C*_max_), time to reach *C*_max_ (*t*_max_), and the truncated area under the time–concentration curve (AUC_last_).

## Results

### General

A total of 16 patients with advanced solid tumours were enrolled in this study (Table 1). Most were considered heavily pre-treated, with a median of three prior chemotherapy regimens (range 0–10 regimens). None of the three patients entered at dose level 1 experienced DLT in cycle 1, allowing escalation to dose level 2. At dose level 2, none of the first 6 patients experienced DLT in cycle 1; thus, this cohort was expanded (Table 2) to 13 patients (one patient who experienced tumour progression on day 8 of cycle 1 was not fully evaluable for safety). Among 12 patients evaluable for toxicity at dose level 2, 2 patients experienced DLTs; one with grade 3 neutropenia, the other with grade 3 neutropenia and grade 2 thrombocytopenia. The toxicities recovered within 2 weeks but were interpreted as *de facto* DLTs, because the paclitaxel dose was immediately reduced by 25% for subsequent cycles in conformity with the manufacturer's recommendations. Median duration of everolimus and paclitaxel at dose level 2 was three cycles; three patients completed six cycles, including two patients with metastatic breast cancer who continued to receive single-agent everolimus for 8 and 18 weeks after paclitaxel discontinuation.

*Safety* No toxicity-related death was reported. The most frequently reported adverse events of any grade irrespective of causality occurring in any treatment cycle were asthenia (81.3%), neutropenia (62.5%), alopecia and anaemia (each 43.8%), and stomatitis, nausea, and pyrexia (each 37.5%). Grade 3/4 adverse events reported in 11 patients included 1 patient with an unrelated grade 4 necrosis of the toe. The most frequently reported drug-related toxicities (Table 3) included neutropenia (56.3%), alopecia and asthenia (each 43.8%), and anaemia and stomatitis (each 37.5%). Thirteen drug-related grade 3 toxicities reported in eight patients, all at dose level 2, included neutropenia in six patients (37.5%), leucopenia in two patients (12.5%), and anaemia, thrombocytopenia, asthenia, stomatitis, and increased alanine aminotransferase levels in individual patients. No drug-related grade 4 toxicity was reported. Serious adverse events occurred in six patients, four directly linked with tumour progression; the other two included a case of grade 3 erysipelas and grade 1 neutropenia in a patient with skin melanoma, and grade 3 interstitial pneumonitis at cycle 6 in another with metastatic breast cancer. In the latter patient, interstitial pneumonitis was confirmed by bronchoscopy and was reversible under treatment with corticosteroids.

*Pharmacokinetics* Blood samples were obtained from 11 patients, including 3 patients treated at dose level 1 and 8 patients at dose level 2. Three patients at dose level 2 did not provide blood samples for determination of everolimus pharmacokinetics. In addition, one patient treated at dose level 2 displayed a threefold increase of everolimus AUC that was found to be related to concomitant medication with fluconazole, invalidating any possible assessment of drug interaction between paclitaxel and everolimus.

**Figure 1:**
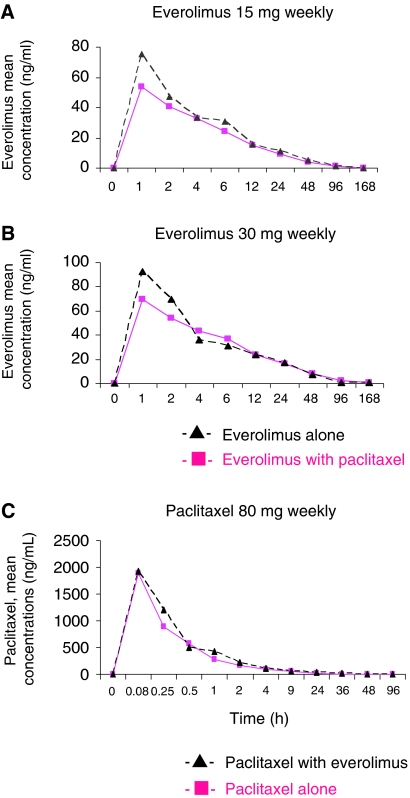
Pharmacokinetic profiles of everolimus in combination with paclitaxel. (**A**) Mean plasma concentration of everolimus following oral administration of 15 mg everolimus alone (*n*=3) or combined with a 1-h infusion of paclitaxel (80 mg m^−2^). (**B**) Mean plasma concentration of everolimus following oral administration of 30 mg everolimus alone (*n*=4) or combined with a 1-h infusion of paclitaxel (80 mg m^−2^). (**C**) Mean plasma concentration of paclitaxel following a 1-h infusion of 80 mg m^−2^ paclitaxel alone (*n*=7) or combined with 15 and 30 mg everolimus.

Representative pharmacokinetic curves of patients treated at dose level 2 are shown in Figure 1, and the main pharmacokinetic parameters of everolimus and paclitaxel are shown in Table 4. As shown, no meaningful change in *t*_max_, *C*_max_, and AUC_last_ of paclitaxel and everolimus was detectable when the drugs were given in combination.

*Response to therapy* No objective response was observed. Sustained tumour stabilisation for more than 4 months was observed in six patients, including those with breast cancer (two patients), ovarian carcinoma (two patients), thyroid carcinoma (one patient), and liposarcoma (one patient). In two patients with metastatic breast cancer treated at dose level 2, tumour stabilisation that lasted >6 months exceeded the duration of tumour stabilisation previously recorded with docetaxel therapy (3.7 and 3.5 months).

## Discussion

Genetic defects in cancer cells expressed as the constitutive activation of growth factor signalling pathways provide stimulation for continued cell growth, survival, and resistance to chemotherapy. In tumours addicted to this growth factor stimulation, chemotherapy combined with mTOR inhibition has shown synergistic anti-tumour effects. For example, data have shown that rapamycin and everolimus can enhance the anti-tumour activity of paclitaxel (O'Reilly *et al*, 2003; Mondesire *et al*, 2004; Faried *et al*, 2006; Aissat *et al*, 2007; Haritunians *et al*, 2007) in a manner related in occurrence and extent to the tumour, dose, and interestingly, the sequence of administration of the mTOR inhibitor and paclitaxel (O'Reilly *et al*, 2003; Mondesire *et al*, 2004; Faried *et al*, 2006; Aissat *et al*, 2007). *In vitro*, paclitaxel treatment before mTOR inhibition produced greater enhancement of apoptosis than treatment after or simultaneously with the mTOR inhibitor, which may reflect a conflict between the slowing of the cell cycle through the G_1_–S transition by mTOR inhibition and the requirement that cells be in G_2_–M transition for paclitaxel-induced apoptosis (Mondesire *et al*, 2004; Aissat *et al*, 2007). Alternatively or in addition, enhancement of cytotoxicity may directly involve p70S6 kinase, which is activated by mTOR. Direct inactivation of p70S6 kinase in mitotic breast and ovarian cancer by paclitaxel has been proposed as a mechanism through which paclitaxel may exert an anti-tumour effect (Le *et al*, 2003). p70S6 kinase inactivates the pro-apoptotic factor BAD; mTOR inhibitors or paclitaxel may promote apoptosis by preventing this inactivation. Consistent with these mechanisms, it has been shown that breast cancer cells resistant to the growth inhibiting effects of rapamycin are also resistant to its chemosensitising effects (Mondesire *et al*, 2004), and sequence-dependent enhancement of paclitaxel cytotoxicity has also been seen with gefitinib and trastuzumab, agents that like mTOR inhibitors inhibit growth factor signal transduction (Lee *et al*, 2002; Magné *et al*, 2002). However, in another study with cervical cancer cells, treatment with rapamycin preceding paclitaxel produced the more favourable result (Faried *et al*, 2006), suggesting that sequence dependency may be influenced by different mechanisms in other types of cancer. Inhibition of protein synthesis by mTOR inhibition may additionally contribute to apoptosis by preventing repair of paclitaxel-induced cell damage (Aissat *et al*, 2007). The extent to which sequence dependency translates *in vivo* is also not clear. In one study, simultaneous treatment with everolimus and paclitaxel produced the greatest enhancement of paclitaxel activity (O'Reilly *et al*, 2003), whereas in another study with xenografts derived from breast cancer cells in which sequence effects were seen *in vitro*, no sequence effects were observed *in vivo* (Mondesire *et al*, 2004). In this trial based on our earlier work (O'Reilly *et al*, 2003), everolimus was administered weekly immediately before paclitaxel infusion to avoid or minimise scheduling effects.

In a report on the pharmacodynamics of weekly paclitaxel in cervical cancer patients, paclitaxel could be detected in tissue for up to 6 days and could induce apoptosis for up to 1 week after treatment, but after 2 weeks, it could no longer be detected in the tumour (Mori *et al*, 2007). The selection of 30 mg per week as the dosage for everolimus was based on the results of a phase I study in which single doses of everolimus of up to 30 mg had been shown to be satisfactorily safe and capable of inhibiting a biomarker indicative of target activity (S6K1 activity in peripheral blood mononuclear cells) (O’Donnell *et al*, 2008). Furthermore, based on the assumption that to be efficacious, the drug should achieve target inhibition that is at least as great as that associated with efficacy in the rat model (Tanaka *et al*, 2008), and accepting the modelling assumption of a similar pharmacokinetic–pharmacodynamic relationship for tumour in patients and in rats, 20–30 mg was identified as the minimal weekly dose. In addition, immunohistochemical analyses in paired tumour biopsies taken before and during weekly dosing (20, 50, or 70 mg) confirmed inhibition of S6K1 at doses ⩾20 mg (Tabernero *et al*, 2008). Consequently, in this study, a weekly dose of everolimus, 15 or 30 mg for 3 weeks followed by a 1-week rest period, was selected as likely to allow adequate inhibition of mTOR signalling, with a period of recovery between doses in the event that relief of mTOR inhibition or paclitaxel exposure enhances the apoptosis-inducing effects of this combination. A lower paclitaxel dose intensity with the 3-weeks-on, 1-week-off schedule compared with a continuous weekly schedule may compromise anti-tumour activity, although paclitaxel regimens with doses of 70–100 mg m^−2^ administered for 3 weeks of each 4-week cycle have been used in combination with cytotoxic agents in patients with advanced cancers with encouraging evidence of anti-tumour activity and acceptable tolerability (Perez *et al*, 2005; Yeh *et al*, 2005; Ramalingam *et al*, 2006; Stathopoulos *et al*, 2007). With this regimen, we have shown that the toxicity of paclitaxel was not enhanced in combination with everolimus.

The most commonly reported drug-related adverse events were haematological toxicity, alopecia, asthenia, and stomatitis. Interstitial pneumonitis has been recognised as being associated with sustained treatment with rapamycin and other rapamycin derivatives (Pham *et al*, 2004; El-Maraghi *et al*, 2006). Pulmonary toxicity is a potential toxicity associated with the use of newer anti-neoplastic agents (Vahid and Marik, 2008). In this study, interstitial pneumonitis was of short duration and recovered under corticosteroid therapy. Interestingly, there was no apparent pharmacokinetic drug interaction between everolimus and paclitaxel. Although both drugs are metabolised by CYP450-3A, paclitaxel may be preferentially metabolised by CYP2C8. Further, steroids used in paclitaxel therapy such as dexamethasone, a weak inducer of CYP3A4, did not appear to affect everolimus.

Everolimus and paclitaxel may be co-administered with manageable toxicity. Everolimus 30 mg can be safely administered with paclitaxel 80 mg on a weekly schedule with dosing for 3 weeks on 4-week cycles for several months in human malignancies. No pharmacokinetic interactions were apparent, and neutropenia was the only toxicity considered dose limiting. It is noteworthy that previous attempts to combine mTOR inhibitors with cytotoxic agents were associated with an increased incidence of toxicity that included dose-limiting diarrhoea and stomatitis with 5-fluorouracil (Punt *et al*, 2003) and thrombocytopenia with gemcitabine (Pacey *et al*, 2004). In this regard, the feasibility of combining everolimus with paclitaxel at clinically effective doses of each agent and the delayed tumour progression observed in this heavily pre-treated population warrant further investigation in paclitaxel-sensitive tumours. Preliminary results of a phase I study combining everolimus and paclitaxel with trastuzumab in patients with trastuzumab-resistant breast cancer have been encouraging, in that the combination was adequately tolerated with a high rate of response (46%) in a population pre-treated with both trastuzumab and taxanes (André *et al*, 2008).

## Figures and Tables

**Table 1:** Patient characteristics

*Age, years*	
Median	54.5
Range	33–69
	
*Sex,* n	
Female	13
Male	3
	
*Race,* n	
Caucasian	15
Black	1
	
*WHO performance status,* n	
0	4
1	10
2	2
	
*Primary site,* n	
Breast	5
Ovarian	3
Angiosarcoma	2
Melanoma	1
Other^a^	5
	
*Stage,* n	
IV	15
Unknown	1
	
*Time since diagnosis*, n(%)	
<1 year	1 (6.3)
1–<2 years	3 (18.8)
⩾3 years	2 (12.5)
2–<3 years	10 (62.5)
	
*Prior therapy,* n	
Taxanes	11
Radiation	8
Other chemotherapy	15
	
*Prior chemotherapy regimens,* n	
1	2
2	4
>2	9

WHO=World Health Organization.

a One each in dose level 2: thyroid carcinoma, mixed testicular (mature embryonic and immature teratoma) cancer, abdominal liposarcoma, vaginal cancer, cholangiocarcinoma.

**Table 2:** Treatment administration by dose level

	**Dose level 1 everolimus 15 mg**	**Dose level 2 everolimus 30 mg**	**All**
Treated, *n*	3	13	16
			
*Discontinued,* n			
Due to AEs	0	1	1
Due to PD, tumour complications	3	11	14
For other reason	0	1	1
			
*Cycles completed,*^a^ n			
0	0	1	1
1	0	1	1
2	1	3	4
3	0	4	4
4	2	1	3
5	0	0	0
6^b^	0	3	3
Mean everolimus treatment duration, weeks	12.6	13.8	13.6
Median everolimus treatment duration, weeks	14.1	11.9	12.5
Range, weeks	9–15	1–41	1–41
Dose interruptions in any cycle, *n* (%)	1 (33.3)	7 (53.8)	8 (50.0)

AE=adverse event; PD=progressive disease.

a Cycles completed refers to the number of combined everolimus with paclitaxel 4-week cycles.

b Among the three patients completing six cycles of everolimus 30 mg with paclitaxel therapy, the customary maximum for paclitaxel, two patients with metastatic breast cancer continued to receive everolimus for 8 and 18 weeks.

**Table 3:** Drug-related toxicity of everolimus in combination with paclitaxel (reported in >1 patient or of grade 3)

	**Dose level 1 everolimus 15 mg**		**Dose level 2 everolimus 30 mg**		**All**	
	***n*=3**		***n*=13**		***N*=16**	
	**All**	**Grade 3**	**All**	**Grade 3**	**All**	**Grade 3**
Any	3	—	11	8	14	8
Neutropenia	2	—	7	6	9	6
Alopecia	1	—	6	—	7	—
Asthenia	1	—	6	1	7	1
Anaemia	2	—	4	1	6	1
Stomatitis	1	—	5	1	6	1
Leucopenia	1	—	4	2	5	2
Myalgia	1	—	4	—	5	—
Paresthesia	1	—	3	—	4	—
Thrombocytopenia	0	—	4	1	4	1
Arthralgia	0	—	3	—	3	—
Erythema	0	—	3	—	3	—
Nausea	2	—	1	—	3	—
Skin lesions	0	—	3	—	3	—
Abdominal pain (upper)	1	—	1	—	2	—
Diarrhoea	1	—	1	—	2	—
Headache	1	—	1	—	2	—
Pruritus	0	—	2	—	2	—
Pyrexia	1	—	1	—	2	—
Vomiting	1	—	1	—	2	—
ALT increase	0	—	1	1	1	1

ALT=alanine aminotransferase.

**Table 4:** Pharmacokinetic parameters of everolimus and paclitaxel

	**Dose level 1**				**Dose level 2**			
	**Everolimus (15 mg)**		**Paclitaxel (80 mg m^−2^)**		**Everolimus (30 mg)**		**Paclitaxel (80 mg m^−2^)**	
	**With paclitaxel**	**Alone**	**With everolimus**	**Alone**	**With paclitaxel**	**Alone**	**With everolimus**	**Alone**
	***n*=3**	***n*=3**	***n*=3**	***n*=3**	***n*=7**	***n*=4**	***n*=7**	***n*=4**
*t*_max_ (h)	1.0 (1–4)	1.0 (1–1)	0.08 (0.08–0.08)	0.08 (0.08–0.08)	1.0 (1–4)	1.0 (1–1)	0.08 (0.08–0.25)	0.08 (0.08–0.25)
*C*_max_ (ng ml^−1^)	57.9±35.8	75.2±37.4	1131.7±484.6	2070.0±701.7	94.5±60.1	93.0±28.6	2160.0±684.6	2025.0±691.8
AUC_last_ (ng h m^−1^)	791.8±346.5	954.9±335.8	1494.1±383.6	2051.0±515.2	1667.8±1000.0	1433.6±1119.8	3913.5±1456.5	3710.1±1575.6
*t*_1/2_ (h)	24.6±3.6	29.5±8.5	7.6±3.6	8.3±2.4	27.4±5.6	26.9±7.1	19.7±8.9	12.8±1.8

*t*_max_ values are median (range), the other parameters values are mean (s.d.).
